# Absolute beta power in exercisers and nonexercisers in preparation for the oddball task

**DOI:** 10.1055/s-0044-1791518

**Published:** 2024-10-02

**Authors:** Marcos Machado, Renato Fonseca, Giovanna Zanchetta, Carlos Amoroso, Alexandre Vasconcelos, Élida Costa, Eduardo Nicoliche, Mariana Gongora, Marco Orsini, Renan Vicente, Silmar Teixeira, Henning Budde, Mauricio Cagy, Bruna Velasques, Pedro Ribeiro

**Affiliations:** 1 Universidade Federal do Rio de Janeiro, Escola de Educação Física e Desportos, Rio de Janeiro RJ, Brazil.; 2 Universidade Federal do Rio de Janeiro, Instituto de Psiquiatria, Rio de Janeiro RJ, Brazil.; 3 Universidade Federal Fluminense, Hospital Universitário Antônio Pedro, Niterói RJ, Brazil.; 4 Universidade Federal do Piauí, Departamento de Fisioterapia, Teresina PI, Brazil.; 5 Medical School Hamburg, Faculty of Human Sciences, Hamburg, Germany.; 6 Reykjavik University, Department of Sport Science, Reykjavik, Iceland.; 7 Universidade Federal do Rio de Janeiro, Departamento de Engenharia Biomédica, Rio de Janeiro RJ, Brazil.

**Keywords:** Exercise, Resistance Training, Executive Function, Frontal Lobe, Attention, Electroencephalography, Exercício Físico, Treinamento Resistido, Função Executiva, Lobo Frontal, Atenção, Eletroencefalografia

## Abstract

**Background** High levels of physical conditioning are associated with improvements in cognitive performance. In this sense, electroencephalographic (ECG) correlates are used to investigate the enhancing role of physical exercise on executive functions. Oscillations in the β frequency range are proposed to be evident during sensorimotor activity.

**Objective** To investigate the ECG changes influenced by aerobic and resistance exercises performed in an attention task by analyzing the differences in absolute β power in the prefrontal and frontal regions before, during, and after the oddball paradigm in practitioners and nonpractitioners of physical exercise.

**Methods** There were 15 physical activity practitioners (aged 27 ± 4.71) and 15 nonpractitioners (age 28 ± 1.50) recruited. A two-way analysis of variance (ANOVA) was implemented to observe the main effect and the interaction between groups and moments (rest 1, pre-stimulus, and rest 2).

**Results** An interaction between group and moment factors was observed for Fp1 ( *p* < 0.001); Fp2 ( *p* = 0.001); F7 ( *p* < 0.001); F8 ( *p* < 0.001); F3 ( *p* < 0.001); Fz ( *p* < 0.001); and F4 ( *p* < 0.001). Electrophysiological findings clarified exercisers' specificity and neural efficiency in each prefrontal and frontal subarea.

**Conclusion** Our findings lend support to the current understanding of the cognitive processes underlying physical exercise and provide new evidence on the relationship between exercise and cortical activity.

## Resumo

**Antecedentes** Níveis elevados de condicionamento físico estão associados a melhorias no desempenho cognitivo. Nesse sentido, correlatos eletroencefalográficos são utilizados na investigação do papel aprimorador do exercício físico sobre as funções executivas. Tem sido proposto que as oscilações na faixa de frequência β são evidenciadas durante a atividade sensório-motora.

**Objetivo** Investigar as alterações eletroencefalográficas influenciadas por exercícios aeróbio e resistido realizados em uma tarefa atencional analisando as diferenças da potência absoluta de β nas regiões pré-frontal e frontal antes, na preparação e depois do paradigma oddball em praticantes e não praticantes de exercício físico.

**Métodos** Foram recrutados 15 praticantes de atividade física (idade 27 ± 4.71) e 15 não praticantes (idade 28 ± 1.50). Uma análise de variância (ANOVA) de duas vias foi implementada para observação do efeito principal e a interação entre os grupos e os momentos (repouso 1, pré-estímulo e repouso 2).

**Resultados** Uma interação entre os fatores grupo e momento para Fp1 ( *p* < 0,001); Fp2 ( *p* = 0,001); F7 ( *p* < 0,001); F8 ( *p* < 0,001); F3 ( *p* < 0,001); Fz ( *p* < 0,001); e F4 ( *p* < 0,001) foi observada. Os achados eletrofisiológicos esclareceram a especificidade e a eficiência neural dos praticantes de exercício físico em cada subárea pré-frontal e frontal.

**Conclusão** Nossos achados promovem o entendimento atual dos processos cognitivos subjacentes ao exercício físico e acrescentam novas evidências sobre a relação exercício e atividade cortical.

## INTRODUCTION

Physical exercise has become an important public health tool, primarily due to its impact on conditioning, cardiorespiratory and physiological parameters. 1 However, while the dynamics and regulatory mechanisms of the body's systems and organs through movement are well documented, understanding of cognitive functioning after physical exercise remains incomplete. 2 Evidence suggests that aerobic and resistance exercises, or combinations of both have facilitative effects on global cognitive development at all ages, 3 4 5 enhancing cognitive functions such as attention, which can be observed by behavioral differences in processing speed and reaction time. 6 7 8

For example, cardiorespiratory fitness has been associated with cognitive abilities that are highly dependent on the frontal lobe in individuals over the age of 55; 9 running improved performance in cognitive tasks associated with neuronal plasticity in the prefrontal cortex; 10 an increase in P300 amplitude was observed after a 15-minute walk outdoors; 11 a single session of aerobic and resistance exercises is associated with improved executive function performance, especially attention allocation. 12 13 Additionally, a recent review analyzed that resistance exercises caused brain changes, especially in the frontal lobe, which were accompanied by executive function improvements; 14 also, exercises contributed to improving the functional plasticity of response inhibition processes in the cerebral cortex; 15 and high-intensity aerobic exercises improved brain activation, reflecting sustained attention during task performance. 16

Electrophysiological correlates indicate that cognitive processes are enhanced over time and that a greater attentive preparatory state is related to higher levels of physical conditioning. 7 Quantitative electroencephalography (EEGq), through its high temporal resolution, has become a suitable tool for examining the electrocortical dynamics underlying neural processing by physical exercise. 17 18 It has been proposed that neural oscillations in the β frequency range (13–30 Hz) are evidenced during sensorimotor activity. 19 20 21 Other studies have observed that an increase in β after exercise is associated with greater cortical activation and cognitive enhancement. 22 23 24 25 26 However, although the association between β and motor activity is well-defined, the frequency's functional significance is still debated.

Therefore, there has been an increasing effort to understand how physical exercise affects cognitive functions and cortical activation. However, few studies have reported the effects of the relationship between physical exercise and cognitive functioning on cortical electrophysiological activity during an attention-heavy movement preparation task.

This study aims to investigate the electrophysiological changes influenced by aerobic and resistance exercises in attentional tasks, using the oddball paradigm. Specifically, we aimed to examine changes in absolute β power in the prefrontal and frontal regions among nonexercisers and exercisers. We hypothesize that the group of exercisers will present a greater efficiency in cortical processing and, consequently, a decreased β activity related to the effect of physical conditioning.

## METHODS

### Sample

We recruited 15 physical activity practitioners (aged 27 ± 4.71) and 15 nonpractitioners (aged 28 ± 1.50), of both sexes, right-handed. The practitioners performed approximately 30 minutes of strength training with both multi and single-joint exercises (50–90% loads of one repetition maximum; 60–90 seconds rest) and 30 minutes of aerobic training (running) at an intensity of 70 to 90% of the maximum heart rate, three times a week for 24 weeks, without interruption. In comparison, nonpractitioners were not physically active in the last 6 months. We utilized the Edinburgh inventory 27 to identify and exclude left-handed individuals from the experiment. All subjects signed an informed consent form and were aware of the experimental protocol. The research was approved by the Ethics Committee of the Federal University of Rio de Janeiro (CAAE: 94638124.2.000.3422), according to the Human Research Ethics Criteria included in the Declaration of Helsinki of 1964.

### Experimental procedure

The participants sat comfortably in a chair with armrests adjusted according to individuals' height to minimize any muscular artifact during EEG. The room used to capture the signal was sound-protected. To perform data acquisition, we reduced brightness to minimize sensory interference during the experiment. The experimental design was divided into three parts: first, we submitted the participants to an EEG record, resting for 3 minutes with eyes open; then, they executed the paradigm (explained below) simultaneously with the EEG record; and after the task, they were submitted to another EEG record at rest for 3 minute, with eyes open.

The oddball paradigm 28 consists of two stimuli presented randomly, one of which occurs infrequently. The subjects must discriminate target (infrequent) from nontarget or standard stimuli (frequent). In the present experiment, target stimuli corresponded to a square, and nontarget stimulus to a circle. We instructed subjects to keep their eyes fixed on the center of the screen and respond as quickly as possible to the target stimulus by pressing a button on a joystick (Quickshot Crystal CS4281). Each stimulus lasted 2.5 seconds, with the same interval time between stimuli, with the screen turned off. We submitted each participant to six blocks of ten trials. In other words, the square was presented ten times in each stage.

### Electroencephalography data acquisition

We recorded the signal acquisition using the 20-channel Braintech-3000 EEG system (EMSA Medical Instruments, Rio de Janeiro, RJ, Brazil). Additionally, two more electrodes were positioned on the earlobes, set as reference points, yielding 20 monopole derivations (using Fpz as a ground electrode). The signal corresponding to each EEG derivation resulted from the electric potential difference between each electrode and the preestablished reference (earlobes). We calculated the impedance levels of each EEG electrode and kept them below 5 kΩ. The data acquired had a total amplitude of less than 100 μV. The EEG signal was amplified, with a gain of 22,000, analogically filtered between 0.1 (high-pass) and 100 Hz (low-pass), and sampled at 240 Hz, Notch (60 Hz).

### Electroencephalography data processing

Visual inspection and independent component analysis (ICA) were used to quantify reference-free data by removing possible sources of task-induced artifacts. We excluded data from individual electrodes exhibiting loss of contact with the scalp or high impedances (> 5kΩ), as well as data from single-trial epochs that showed excessive movement artifact (± 100μV). Then, ICA was applied to identify and remove any artifacts after the initial visual inspection. Independent components resembling an eye blink or muscle artifact were also removed. The remaining components were then projected back onto the scalp electrodes by multiplying the input data by the inverse matrix of the spatial filter coefficients, derived from ICA using established procedures. This data were then reinspected for residual artifacts using the rejection criteria described above. We reduced quantitative EEG parameters to 4 seconds before the oddball paradigm joystick button press, that is, the moment preceding index finger movement.

### Statistical analysis

The Levene and Shapiro-Wilk tests previously verified the normality and homoscedasticity of the data. Afterward, a two-way analysis of variance (ANOVA) was used between the group and the moments, aiming to explore the interaction. Consequently, a repeated-measures ANOVA was applied to identify the EEG-β power differences for moments within each group. We used the Mauchly test criteria to evaluate the sphericity hypothesis and the Greenhouse-Geisser (Gε) procedure to correct freedom degrees. We analyzed the interactions with multiple comparisons by the Bonferroni post hoc. We estimated the size effect as partial squared eta (ƞ2p), and calculated the statistical power and the 95% confidence interval for dependent variables. We interpreted the magnitude of the effect by using the recommendations suggested by Hopkins et al.: 0.0 = trivial; 0.2 = small; 0.6 = moderate; 1.2 = large; 2.0 = very large; and 4.0 = almost perfect. 29 To detect the real difference in the population, we interpreted a statistical power from 0.8 to 0.9 as high power [R]. 30 Thus, the *p* -value was divided by the number of cortical regions analyzed, from a total of 7 ( *p* = 0.0071). The probability of 5% was adopted in all analyses ( *p* ≤ 0.05).

## RESULTS

**Figure 1:**
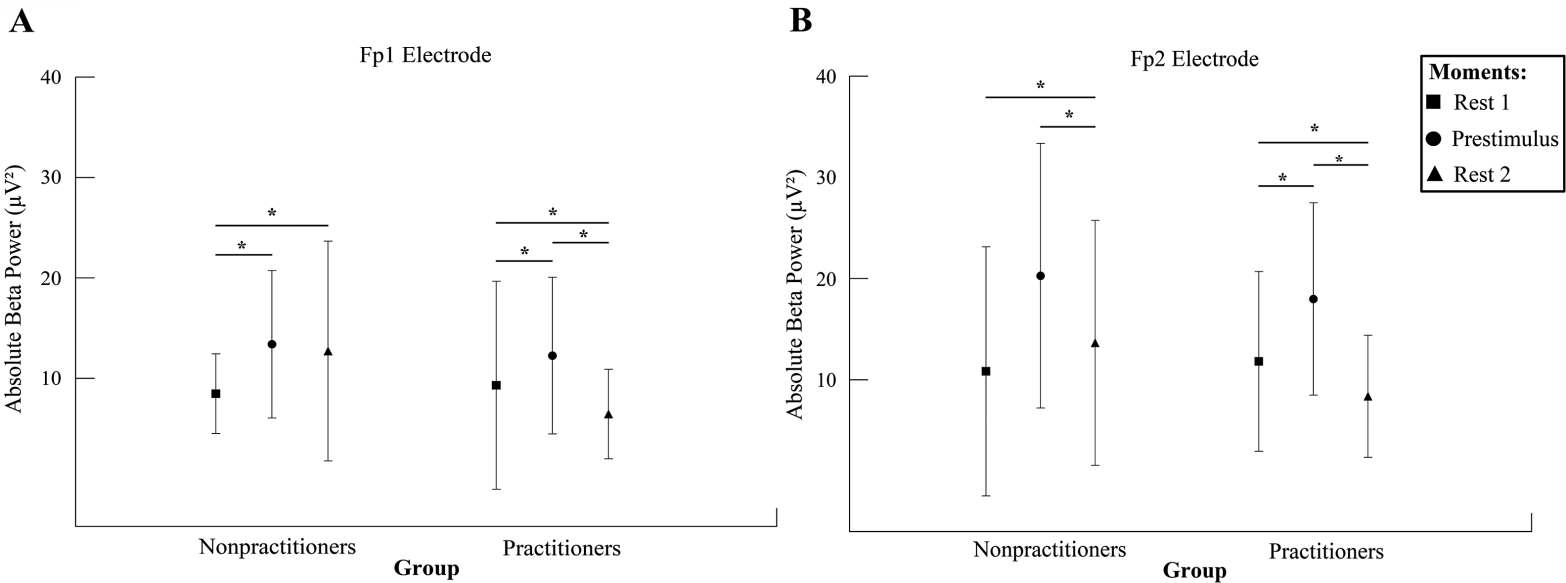
The mean and standard deviation of absolute β power (μV ^2^ ). ( **A** ) Left frontopolar cortex (Fp1) interaction between group and moments (nonpractitioners vs. practitioners vs. rest 1 vs. prestimulus vs. rest 2; *p* < 0.001). The repeated measures ANOVA demonstrated a significant difference for a moment rest 2 compared with moment prestimulus between groups ( *p* < 0.001). ( **B** ) The right frontopolar cortex (Fp2) interaction between group and moments (nonpractitioners vs. practitioners vs. rest 1 vs. prestimulus vs. rest 2; *p* < 0.001). The repeated measures ANOVA demonstrated a significant difference for prestimulus compared with rest 1, and for rest 2 compared with rest 1, between groups ( *p* < 0.001). Bonferroni post hoc comparisons are highlighted by asterisks.

There was an interaction between the group and moment factors for Fp1 ( *F*_(2.374)_ = 22.041; *p* < 0.001; *n* 2 *p* = 0.105; power = 99.9%; Figure 1A ). The Mauchly test indicated that sphericity was considered ( *p* = 0.140). The post hoc test showed that, in the group of nonexercisers, resting time 1 was different from prestimulus ( *p* < 0.001) and resting time 2 ( *p* < 0.001). For practitioners, resting time 1 was different from prestimulus ( *p* = 0.027) and resting time 2 ( *p* = 0.001); also, prestimulus was different from resting time 2 ( *p* < 0.001).

Concerning Fp2, we saw an interaction between the factors group and time ( *F*_(2.342)_ = 7.392; *p* = 0.001; *n* 2 *p* = 0.041; power = 93.9%; Figure 1B ). The Mauchly test indicated that sphericity was considered ( *p* = 0.452). The post hoc test showed that, in the group of nonexercisers, resting time 1 was different from resting time 2 ( *p* < 0.001); and prestimulus time was different from resting time 2 ( *p* < 0.001). For practitioners, resting time 1 was different from prestimulus ( *p* < 0.001) and resting time 2 ( *p* < 0.001); also, prestimulus was different from resting time 2 ( *p* < 0.001).

**Figure 2:**
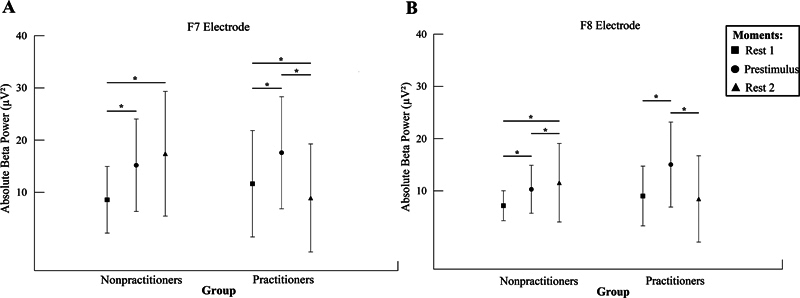
The mean and standard deviation of absolute β power (μV ^2^ ). ( **A** ) Left inferior prefrontal gyrus (F7) interaction between groups and moments (nonpractitioners vs. practitioners vs. rest 1 vs. prestimulus vs. rest 2; *p* < 0.001). The repeated measures ANOVA demonstrated a significant difference for prestimulus compared with rest 2, and a significant difference for rest 2 compared with rest 1, between groups ( *p* < 0.001). ( **B** ) The right prefrontal gyrus (F8) interaction between groups and moments (nonpractitioners vs. practitioners vs. rest 1 vs. prestimulus vs. rest 2; *p* < 0.001). The repeated measures ANOVA demonstrated a significant difference for rest 2 compared with prestimulus ( *p* < 0.001). The Bonferroni post hoc comparisons are highlighted by asterisks.

We found an interaction between the group and moment factors for F7 ( *F*_(1.920, 359.110)_ = 42.345; *p* < 0.001; *n* 2 *p* = 0.185; power = 99.9%; Figure 2A ). The Mauchly test indicated that sphericity was violated ( *p* = 0.019). Therefore, the degrees of freedom were adjusted (ε = 0.960). The post hoc test showed that, in the group of nonexercisers, resting time 1 was different from prestimulus ( *p* < 0.001) and resting time 2 ( *p* < 0.001). For practitioners, resting time 1 was different from prestimulus ( *p* = 0.027) and resting time 2 ( *p* = 0.001); also, prestimulus was different from resting time 2 ( *p* < 0.001).

There was also an interaction between the factors group and time for F8 ( *F* [1.764, 333.468] = 33.388; *p* < 0.001; *n* 2 *p* = 0.150; power = 99.9%; Figure 2B ). The Mauchly test indicated that sphericity was violated ( *p* < 0.001). Therefore, the degrees of freedom were adjusted (ε = 0.882). The post hoc test showed that, in the group of nonpractitioners, resting moment 1 was different from prestimulus ( *p* < 0.001) and resting moment 2 ( *p* < 0.001); and prestimulus moment was different from resting moment 2 ( *p* = 0.038). For practitioners, resting time 1 was different from prestimulus ( *p* < 0.001); also, prestimulus was different from resting time 2 ( *p* < 0.001).

**Figure 3:**
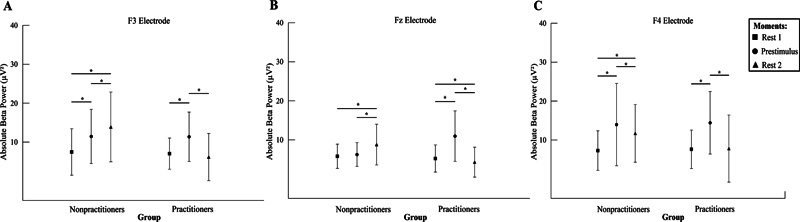
The mean and standard deviation of absolute β power (μV ^2^ ). ( **A** ) Left anterior frontal cortex (F3) interaction between group and moments (nonpractitioners vs. practitioners vs. rest 1 vs. prestimulus vs. rest 2; *p* < 0.001). The repeated measures ANOVA demonstrated a significant difference for rest 2 compared with prestimulus between groups ( *p* < 0.001). ( **B** ) The midline anterior frontal cortex (Fz) interaction between group and moments (nonpractitioners vs. practitioners vs. rest 1 vs. prestimulus vs. rest 2; *p* < 0.001). The repeated measures ANOVA demonstrated a significant difference for prestimulus compared with rest 2 ( *p* < 0.038), and a significant difference for rest 2 compared with rest 1 between groups ( *p* < 0.007). ( **C** ) The right anterior frontal cortex (F4) interaction between group and moments (nonpractitioners vs. practitioners vs. rest 1 vs. prestimulus vs. rest 2; *p* < 0.001). The repeated measures ANOVA demonstrated a significant difference for prestimulus compared with rest 2, between groups ( *p* < 0.001). Bonferroni post hoc comparisons are highlighted by asterisks.

We observed an interaction between the group and moment factors for F3 ( *F*_(1.904, 359.831)_ = 45.386; *p* < 0.001; *n* 2 *p* = 0.194; power = 99.9%; Figure 3A ). The Mauchly test indicated that sphericity was violated ( *p* = 0.008), so the degrees of freedom were adjusted (ε = 0.952). The post hoc test showed that, in the group of nonpractitioners, resting time 1 was different from prestimulus ( *p* < 0.001) and resting time 2 ( *p* < 0.001); also, pre-stimulus was different from resting time 2 ( *p* = 0.007). For practitioners, resting time 1 was different from prestimulus ( *p* < 0.001); also, prestimulus was different from resting time 2 ( *p* < 0.001).

Regarding Fz, there was an interaction between the factors group and time ( *F*_(1.573, 297.313)_ = 106.211; *p* < 0.001; *n* 2 *p* = 0.360; power = 99.9%; Figure 3B ). The Mauchly test indicated that sphericity was violated ( *p* < 0.001), so the degrees of freedom were adjusted (ε = 0.787). The post hoc test showed that, in the group of nonexercisers, resting time 1 was different from resting time 2 ( *p* < 0.001); also, prestimulus was different from resting time 2 ( *p* < 0.001). For practitioners, resting time 1 was different from prestimulus ( *p* < 0.001) and resting time 2 ( *p* = 0.007); also, prestimulus was different from resting time 2 ( *p* = 0.038).

Concerning F4, there was an interaction between the group and moment factors ( *F*_(2.378)_ = 10.397; *p* < 0.001; *n* 2 *p* = 0.052; power = 98.8%; Figure 3C ). The Mauchly test indicated that sphericity was considered ( *p* = 0.051). The post hoc test showed that, in the group of nonexercisers, resting time 1 was different from prestimulus ( *p* < 0.001) and resting time 2 ( *p* < 0.001); also, pre-stimulus was different from resting time 2 ( *p* = 0.023). For practitioners, resting time 1 was different from prestimulus ( *p* = 0.027); also, prestimulus was different from resting time 2 ( *p* < 0.001).

## DISCUSSION

The differences in absolute β power in the prefrontal and frontal regions before, in preparation for, and after the oddball paradigm in nonexercisers and exercisers were analyzed. The investigation of these areas is especially relevant due to their involvement with cognitive functions and motor aspects. 31 We expected a decrease in β, related to the effect of physical conditioning according to the principle of neural efficiency. The results were in line with our hypothesis in the three subregions analyzed: the anterior prefrontal cortex (Fp1 and Fp2), the inferior prefrontal gyrus (F7 and F8), and the frontal eye field (F3, Fz, and F4).

The anterior prefrontal cortex (or frontopolar cortex), located anatomically over the anterior frontal region of the cortex (Brodmann areas 9 and 10), has been associated with complex executive functions that play an important role in task organization, planning, motor representation, and decision-making. 32 33 Our results showed a significant interaction between groups and times for both electrodes (Fp1 and Fp2).

In this sense, the subsequent analysis for Fp1 showed that the differences between the groups are related to resting times 1 and 2, and the prestimulus and resting time 2. The decrease in β observed for the group of exercisers, compared with the nonexercisers, may suggest the idea of neural efficiency in the frontopolar cortex region after a task. 34 A recent study compared β EEG in groups of physical activity practitioners and nonpractitioners during a time-estimation task. 35 The results showed lower β power for the practitioners. The authors suggested that lower β and better performance in the time estimation task may indicate maintenance of attention and neural efficiency. Additionally, a meta-analysis investigated the effects of physical training on executive functions in adults, 36 with the results reporting benefits after physical training, suggesting that training has an enhancing effect on cognition.

Concerning Fp2, further analysis showed that the differences between groups were related to resting time 1 and prestimulus, as well as resting times 1 and 2. The results reveal the mutual participation of the frontopolar cortex during the experiment and reflect an efficient oscillatory mode for practitioners, related to greater aptitude after meeting the demands of the attentional paradigm, represented by a greater state of cortical deactivation. This difference may represent practitioners' more efficient neural networks, which are involved in allocating attention from the start of the experiment. 6

These findings align with previous works highlighting differences in cortical activity underlying physical training. A previous study investigated the speed of information processing in athletes compared with a control group. 37 The results indicated the amount of practice allowed for the development of preparatory activities, which may suggest that the β dynamics indicate that the effects of physical exercise are correlated with cognitive improvement, meaning that tasks set by internal factors are associated with a cognitive effort dependent on executive functions, reflected by β activity.

The inferior prefrontal gyrus has been associated with language, 38 mnemonic processes, 39 and action observation. 40 Previous studies have indicated the involvement of this area in processing semantic language and nonverbal attributes. 41 Our results demonstrated a significant interaction between the groups and time points for both electrodes (F7 and F8).

We also observed a decrease in β in the group of practitioners within the left inferior prefrontal gyrus (F7). This result suggests less cortical activation in the identification, analysis, and attention to information during the experimental paradigm, especially in the hemisphere characterized by analytical processes. 42 The decrease in β can be understood as efficiency during the experimental task, that is, reduced effort and, consequently, a deactivation. A recent study suggested that a single session of moderate-intensity aerobic exercise improves performance in mnemonic discrimination tasks in healthy individuals. 43

As for F8, the subsequent analysis showed that the differences between groups are related to resting times 1 and 2, along with prestimulus and resting time 2. The dynamics of F8 can be explained by the behavior observed in prestimulus and resting time 2. In contrast, the cortical deactivation observed for the group of exercisers can be understood as an efficiency, that is, a decrease in power over the region after the experimental task. 44 Thus, our findings represent an efficient oscillatory β mode related to a greater aptitude for the experimental paradigm's demands, represented by cortical deactivation in the exercisers.

Located anatomically in the premotor cortex (Brodmann area 8), the anterior frontal cortex plays a complex role in various higher functions, such as planning, sustained attention, and motor learning. 45 46 Additionally, this region plays an important role in planning and controlling saccadic eye movements. 47 Our results showed a significant interaction between groups and times for both electrodes (F3, Fz, and F4).

The analysis of the interaction in F3 revealed that the differences between groups are related to resting times 1 and 2, as well as prestimulus and resting time 2. The results observed for the group of practitioners can be understood as a state close to automaticity during the experimental task, favoring energy saving, as observed by the decrease in β.

Decreases in β have been observed in the sensorimotor and frontal areas in young adults after a motor task, probably indicating automaticity in motor control, 48 49 reflecting β's functional role in motor learning processes. A previous study observed a greater increase in β among sedentary individuals compared with the athletic group, suggesting that active individuals were more successful at maintaining focus under stress compared with sedentary individuals. 50

Concerning Fz, further analysis has shown that differences between groups are related across all three moments. The dynamics observed for the exercising group can be explained by sophisticated cortical redistribution, where cortical areas not relevant to the task are disregarded, that is, energy saving. 18 A recent study proposed that exercise involves excitation mechanisms through various neurotransmitter systems to facilitate the processing of implicit information, which deactivates higher-order functions of the cortex to prevent useless processes from compromising the implicit system's functioning during motor execution. 16

Finally, the subsequent analysis in F4 showed that the differences between groups were related at resting times 1 and 2. The results may indicate that the group of practitioners automated the reactions between visual stimulus and imposed response, meaning there was a decrease in β to its initial state after the task. 51 A cross-sectional study showed a relationship between aerobic fitness and neural rhythms in a visuospatial attention task among young adults of high- and low-levels of fitness. The high-fitness participants had faster reaction times and greater β during target processing. These findings indicated that physical fitness may be positively correlated to greater visuospatial attention capacity by modulating attentional processes. 52

## Limitations of this study

- Initially, the electroencephalographic analysis was limited to only 20 channels, providing an adequate spatial sample for future analysis.
- Our findings are quite discrete, suggesting that claims regarding the significance of physical exercise on cognitive functions should be more moderate, and that further analyses with other variables are needed.
- Based on the literature indicating physical exercise as a neuroenhancer, it is essential to consider the use of other protocols in future studies. These may include exploring the potential effects related to cognitive events before and after physical training.

In conclusion, this study investigated the electrophysiological changes influenced by physical exercise during an attentional task. Our findings provide new evidence on the relationship between physical exercise and cortical activity. Thus, we conclude that ECG correlates of the prefrontal and frontal regions promote understanding of the cognitive processes underlying physical exercise and can be used in future interventions and posttraining extrinsic feedback.

## References

[1] Schneider S Brümmer V Abel T Askew C D Strüder H K Changes in brain cortical activity measured by EEG are related to individual exercise preferences Physiol Behav 2009 98 04 447 452 10.1016/j.physbeh.2009.07.010 19643120

[2] Ciria L F Luque-Casado A Sanabria D Holgado D Ivanov P C Perakakis P Oscillatory brain activity during acute exercise: Tonic and transient neural response to an oddball task Psychophysiology 2019 56 05 e13326 10.1111/psyp.13326 30637763 PMC7311047

[3] Lardon M T Polich J EEG changes from long-term physical exercise Biol Psychol 1996 44 01 19 30 10.1016/s0301-0511(96)05198-8 8906355

[4] Berchicci M Pontifex M B Drollette E S Pesce C Hillman C H Di Russo F From cognitive motor preparation to visual processing: The benefits of childhood fitness to brain health Neuroscience 2015 298 211 219 10.1016/j.neuroscience.2015.04.028 25907444

[5] Chow Z-S Moreland A T Macpherson H Teo W P The Central Mechanisms of Resistance Training and Its Effects on Cognitive Function Sports Med 2021 51 12 2483 2506 10.1007/s40279-021-01535-5 34417978

[6] Brockett A T LaMarca E A Gould E Physical exercise enhances cognitive flexibility as well as astrocytic and synaptic markers in the medial prefrontal cortex PLoS One 2015 10 05 e0124859 10.1371/journal.pone.0124859 25938418 PMC4418599

[7] Luque-Casado A Ciria L F Sanabria D Perakakis P Exercise practice associates with different brain rhythmic patterns during vigilance Physiol Behav 2020 224 113033 10.1016/j.physbeh.2020.113033 32598939

[8] Fujiwara H Tsurumi K Shibata M Life Habits and Mental Health: Behavioural Addiction, Health Benefits of Daily Habits, and the Reward System Front Psychiatry 2022 13 813507Doi: 10.3389%2Ffpsyt.2022.813507 35153878 10.3389/fpsyt.2022.813507 PMC8829329

[9] España-Irla G Gomes-Osman J Cattaneo G Associations Between Cardiorespiratory Fitness, Cardiovascular Risk, and Cognition Are Mediated by Structural Brain Health in Midlife J Am Heart Assoc 2021 10 18 e020688 10.1161/JAHA.120.020688 34514813 PMC8649552

[10] Cheron G Petit G Cheron J Brain Oscillations in Sport: Toward EEG Biomarkers of Performance Front Psychol 2016 7 246 26955362 10.3389/fpsyg.2016.00246 PMC4768321

[11] Boere K Lloyd K Binsted G Krigolson O E Exercising is good for the brain but exercising outside is potentially better Sci Rep 2023 13 01 1140 10.1038/s41598-022-26093-2 36670116 PMC9859790

[12] Vonk M Wikkerink S Regan K Middleton L E Similar changes in executive function after moderate resistance training and loadless movement PLoS One 2019 14 02 e0212122 10.1371/journal.pone.0212122 30794593 PMC6386275

[13] Pontifex M B Parks A C Henning D A Kamijo K Single bouts of exercise selectively sustain attentional processes Psychophysiology 2015 52 05 618 625 10.1111/psyp.12395 25523887 PMC4398582

[14] Herold F Törpel A Schega L Müller N G Functional and/or structural brain changes in response to resistance exercises and resistance training lead to cognitive improvements - a systematic review Eur Rev Aging Phys Act 2019 16 10 10.1186/s11556-019-0217-2 31333805 PMC6617693

[15] Liu-Ambrose T Nagamatsu L S Voss M W Khan K M Handy T C Resistance training and functional plasticity of the aging brain: a 12-month randomized controlled trial Neurobiol Aging 2012 33 08 1690 1698 10.1016/j.neurobiolaging.2011.05.010 21741129

[16] Du Rietz E Barker A R Michelini G Beneficial effects of acute high-intensity exercise on electrophysiological indices of attention processes in young adult men Behav Brain Res 2019 359 474 484Doi: 10.1016%2Fj.bbr.2018.11.024 30465815 10.1016/j.bbr.2018.11.024 PMC6320386

[17] Fang Q Fang C Li L Song Y Impact of sport training on adaptations in neural functioning and behavioral performance: A scoping review with meta-analysis on EEG research J Exerc Sci Fit 2022 20 03 206 215 10.1016/j.jesf.2022.04.001 35510253 PMC9035717

[18] Di Muccio F Ruggeri P Brandner C Barral J Electrocortical correlates of the association between cardiorespiratory fitness and sustained attention in young adults Neuropsychologia 2022 172 108271 10.1016/j.neuropsychologia.2022.108271 35595065

[19] Chaire A Becke A Düzel E Effects of Physical Exercise on Working Memory and Attention-Related Neural Oscillations Front Neurosci 2020 14 239 10.3389/fnins.2020.00239 32296302 PMC7136837

[20] John A T Wind J Horst F Schöllhorn W I Acute Effects of an Incremental Exercise Test on Psychophysiological Variables and Their Interaction J Sports Sci Med 2020 19 03 596 612 32874113 PMC7429434

[21] Engel A K Fries P Beta-band oscillations–signalling the status quo? Curr Opin Neurobiol 2010 20 02 156 165 10.1016/j.conb.2010.02.015 20359884

[22] Lee S M Choi M Chun B-O Effects of a High-Intensity Interval Physical Exercise Program on Cognition, Physical Performance, and Electroencephalogram Patterns in Korean Elderly People: A Pilot Study Dement Neurocognitive Disord 2022 21 03 93 102 10.12779/dnd.2022.21.3.93 PMC9340247 35949421

[23] Lin M-A Meng L-F Ouyang Y Resistance-induced brain activity changes during cycle ergometer exercises BMC Sports Sci Med Rehabil 2021 13 01 27Doi: 10.1186%2Fs13102-021-00252-w 33741055 10.1186/s13102-021-00252-w PMC7977282

[24] Dal Maso F Desormeau B Boudrias M-H Roig M Acute cardiovascular exercise promotes functional changes in cortico-motor networks during the early stages of motor memory consolidation Neuroimage 2018 174 380 392 10.1016/j.neuroimage.2018.03.029 29555428

[25] Zaepffel M Trachel R Kilavik B E Brochier T Modulations of EEG beta power during planning and execution of grasping movements PLoS One 2013 8 03 e60060 10.1371/journal.pone.0060060 23555884 PMC3605373

[26] Brümmer V Schneider S Abel T Vogt T Strüder H K Brain cortical activity is influenced by exercise mode and intensity Med Sci Sports Exerc 2011 43 10 1863 1872 10.1249/MSS.0b013e3182172a6f 21364475

[27] Oldfield R C The assessment and analysis of handedness: the Edinburgh inventory Neuropsychologia 1971 9 01 97 113 10.1016/0028-3932(71)90067-4 5146491

[28] Squires N K Squires K C Hillyard S A Two varieties of long-latency positive waves evoked by unpredictable auditory stimuli in man Electroencephalogr Clin Neurophysiol 1975 38 04 387 401 10.1016/0013-4694(75)90263-1 46819

[29] Hopkins W G Marshall S W Batterham A M Hanin J Progressive statistics for studies in sports medicine and exercise science Med Sci Sports Exerc 2009 41 01 3 13 10.1249/MSS.0b013e31818cb278 19092709

[30] Fayers P M Machin D Sample size: how many patients are necessary? Br J Cancer 1995 72 01 1 9 10.1038/bjc.1995.268 7599035 PMC2034119

[31] Pedroso R V Lima-Silva A E Tarachuque P E Fraga F J Stein A M Efficacy of Physical Exercise on Cortical Activity Modulation in Mild Cognitive Impairment: A Systematic Review Arch Phys Med Rehabil 2021 102 12 2393 2401 10.1016/j.apmr.2021.03.032 33932357

[32] Chiang T-C Liang K-C Chen J-H Hsieh C H Huang Y A Brain deactivation in the outperformance in bimodal tasks: an FMRI study PLoS One 2013 8 10 e77408Doi: 10.1371%2Fjournal.pone.0077408 24155952 10.1371/journal.pone.0077408 PMC3796455

[33] Domic-Siede M Irani M Valdés J Perrone-Bertolotti M Ossandón T Theta activity from frontopolar cortex, mid-cingulate cortex and anterior cingulate cortex shows different roles in cognitive planning performance Neuroimage 2021 226 117557 10.1016/j.neuroimage.2020.117557 33189934

[34] Montuori S D'Aurizio G Foti F Executive functioning profiles in elite volleyball athletes: Preliminary results by a sport-specific task switching protocol Hum Mov Sci 2019 63 73 81 10.1016/j.humov.2018.11.011 30503984

[35] da Silva K Curvina M Araújo S Male practitioners of physical activity present lower absolute power of beta band in time perception test Neurosci Lett 2021 764 136210 10.1016/j.neulet.2021.136210 34481000

[36] Chen F-T Etnier J L Chan K-H Chiu P K Hung T M Chang Y K Effects of Exercise Training Interventions on Executive Function in Older Adults: A Systematic Review and Meta-Analysis Sports Med 2020 50 08 1451 1467 10.1007/s40279-020-01292-x 32447717 PMC7376513

[37] Ceyte H Lion A Caudron S Perrin P Gauchard G C Visuo-oculomotor skills related to the visual demands of sporting environments Exp Brain Res 2017 235 01 269 277 10.1007/s00221-016-4793-3 27704155

[38] Schneiders J A Opitz B Tang H The impact of auditory working memory training on the fronto-parietal working memory network Front Hum Neurosci 2012 6 173Doi: 10.3389%2Ffnhum.2012.00173 22701418 10.3389/fnhum.2012.00173 PMC3373207

[39] Yamasaki H LaBar K S McCarthy G Dissociable prefrontal brain systems for attention and emotion Proc Natl Acad Sci U S A 2002 99 17 11447 11451 10.1073/pnas.182176499 12177452 PMC123276

[40] Binkofski F Buccino G The role of ventral premotor cortex in action execution and action understanding J Physiol Paris 2006 99(4-6):396 405 10.1016/j.jphysparis.2006.03.005 16723210

[41] Christophel T B Distributed Visual Working Memory Stores Revealed by Multivariate Pattern Analyses J Vis 2015 15 1407 10.1167/15.12.1407

[42] Ives-Deliperi V L Butler J T Relationship Between EEG Electrode and Functional Cortex in the International 10 to 20 System J Clin Neurophysiol 2018 35 06 504 509 10.1097/WNP.0000000000000510 30387785

[43] Callow D D Pena G S Stark C EL Effects of acute aerobic exercise on mnemonic discrimination performance in older adults J Int Neuropsychol Soc 2023 29 06 519 528 10.1017/S1355617722000492 35968853 PMC10538177

[44] Lim K H-L Pysklywec A Plante M Demers L The effectiveness of Tai Chi for short-term cognitive function improvement in the early stages of dementia in the elderly: a systematic literature review Clin Interv Aging 2019 14 827 839 10.2147/CIA.S202055 31190769 PMC6512568

[45] Lanzilotto M Perciavalle V Lucchetti C A new field in monkey's frontal cortex: premotor ear-eye field (PEEF) Neurosci Biobehav Rev 2013 37 08 1434 1444 10.1016/j.neubiorev.2013.05.010 23727051

[46] Chuang L-Y Huang C-J Hung T-M The differences in frontal midline theta power between successful and unsuccessful basketball free throws of elite basketball players Int J Psychophysiol 2013 90 03 321 328 10.1016/j.ijpsycho.2013.10.002 24126125

[47] Velasques B Bittencourt J Diniz C Changes in saccadic eye movement (SEM) and quantitative EEG parameter in bipolar patients J Affect Disord 2013 145 03 378 385 10.1016/j.jad.2012.04.049 22832171

[48] Nakano H Osumi M Ueta K Kodama T Morioka S Changes in electroencephalographic activity during observation, preparation, and execution of a motor learning task Int J Neurosci 2013 123 12 866 875 10.3109/00207454.2013.813509 23768018

[49] Studer B Koeneke S Blum J Jäncke L The effects of practice distribution upon the regional oscillatory activity in visuomotor learning Behav Brain Funct 2010 6 8 10.1186/1744-9081-6-8 20205755 PMC2822735

[50] Bayazit O Üngür G Neuroelectric responses of sportsmen and sedentaries under cognitive stress Cogn Neurodyn 2018 12 03 295 301Doi: 10.1007%2Fs11571-018-9478-0 29765478 10.1007/s11571-018-9478-0 PMC5943213

[51] Wollenberg L Hanning N M Deubel H Visual attention and eye movement control during oculomotor competition J Vis 2020 20 09 16Doi: 10.1167%2Fjov.20.9.16 10.1167/jov.20.9.16 PMC7521175 32976594

[52] Wang C-H Liang W-K Tseng P Muggleton N G Juan C H Tsai C L The relationship between aerobic fitness and neural oscillations during visuo-spatial attention in young adults Exp Brain Res 2015 233 04 1069 1078 10.1007/s00221-014-4182-8 25537471

